# Effect of Silane Functionalization on the Properties of Polydimethylsiloxane Nanocomposites Reinforced with Reduced Graphene Oxide and Carbon Nanotubes

**DOI:** 10.3390/polym18212650

**Published:** 2026-10-26

**Authors:** Elyff Cargnin, Luís Marcelo Garcia da Silva, Rogerio Ramos de Sousa Junior, Guilherme Elias Saltarelli Garcia, Raquel Bissaco, Dunieskys Roberto González Larrudé, Demetrio Jackson dos Santos

**Affiliations:** 1 School of Engineering, Mackenzie Presbyterian University, São Paulo 01302-907, Brazil; elyff_cargnin@hotmail.com (E.C.); dunieskys.larrude@mackenzie.br (D.R.G.L.); 2 Center of Engineering, Modeling and Applied Social Sciences, Federal University of ABC (UFABC), Santo Andre 09210-580, Brazil; luis.garcia@ufabc.edu.br (L.M.G.d.S.); rogerio.sousa@ufabc.edu.br (R.R.d.S.J.); guilherme.elias@ufabc.edu.br (G.E.S.G.); raquel.bissaco@aluno.ufabc.edu.br (R.B.)

**Keywords:** silane functionalization, nanocomposite, polydimethylsiloxane, graphene, carbon nanotubes

## Abstract

Elastomeric nanocomposites containing carbon nanomaterials have potential for functional applications, although their performance depends on filler–matrix interactions and conductive network formation. This study evaluated the effects of KH550 functionalization and rGO or SWCNT concentration on the thermal, mechanical, and electrical properties of PDMS nanocomposites. Nanomaterial modification was characterized by Raman spectroscopy, SEM, and XPS, while nanocomposites were evaluated by AC conductivity, thermogravimetric analysis, and tensile testing. XPS evidenced changes in surface chemical composition following functionalization. Incorporating up to 2.0 wt.% rGO did not appreciably alter PDMS conductivity, whereas 0.1 wt.% SWCNT increased conductivity from 2.8 × 10^−14^ to 2.7 × 10^−6^ S/m at 0.1 Hz, approximately eight orders of magnitude. SWCNT functionalization reduced conductivity, although increasing filler concentration partially compensated for this effect. At 0.5 wt.% SWCNT, functionalization resulted in 129% higher mean tensile strength and 52% higher mean elongation at break compared with the unfunctionalized nanocomposite. Both nanomaterials modified the thermal behavior of PDMS, while mechanical effects depended on filler type, concentration, and functionalization. These results highlight the need to balance filler–matrix interactions and conductive network formation in PDMS nanocomposites.

## 1. Introduction

Polymer nanocomposites combine a polymer matrix with nanoscale reinforcements to enhance performance and provide functional properties. This is attributed, among other factors, to the high surface area and, depending on their morphology, the high aspect ratio of nanomaterials, which provide a larger polymer–nanomaterial interfacial area and can promote improvements in the mechanical, thermal, and electrical properties of the nanocomposite [1,2]. These characteristics also enable the use of low nanomaterial concentrations [2], contributing to the preservation of the intrinsic characteristics of the polymer matrix. This is particularly relevant for nanocomposites based on elastomeric matrices, which have potential for different technological fields, including flexible and stretchable electronics, where materials must combine high flexibility or deformability with suitable electrical properties [1,2,3,4], with potential applications in systems such as smart textiles, e-skin, sensors for medical applications, soft robots, and artificial muscles [5,6,7].

Elastomeric matrices based on polydimethylsiloxane (PDMS) are widely used in functional nanocomposites because of characteristics such as ease of processing, high flexibility, and thermal stability, which favor their use in deformable systems and their potential application in the field of flexible electronics [8,9,10]. The addition of carbon nanomaterials, such as carbon nanotubes, graphene, and their derivatives, has been an effective strategy for improving electrical and mechanical properties at low concentrations, owing to factors such as their high surface area and aspect ratio [10]. However, for these properties to be effectively achieved, an adequate distribution of the nanomaterials within the polymer matrix and the formation of a conductive network are important. On the other hand, these nanomaterials tend to agglomerate because of van der Waals forces [2,11], which can result in poor dispersion and deteriorate the functional properties of the nanocomposite [12]. Therefore, improving the polymer–nanomaterial interfacial interaction is essential for obtaining nanocomposites that combine flexibility and functional properties.

Several strategies have been employed to improve the dispersibility of nanomaterials within polymer matrices. One approach is the use of carbon nanomaterial derivatives, such as graphene oxide (GO), which contains oxygen-containing functional groups in its structure that can favor its interaction and dispersion in certain solvents and polymer matrices [13,14]. On the other hand, GO exhibits low electrical conductivity because of structural defects and disruption of the conjugated carbon structure [13]. An alternative is the use of reduced graphene oxide (rGO), which exhibits partial recovery of the conjugated structure and, consequently, higher electrical conductivity, although the reduction process can favor the restacking of the graphitic structures [15,16]. Another strategy involves the use of mechanical dispersion methods, such as ball milling or ultrasonication, which can improve nanomaterial dispersion [2,17]. However, excessively severe processing conditions can cause fragmentation or a reduction in nanomaterial dimensions, decreasing their aspect ratio and, consequently, their reinforcing capability and contribution to functional properties. Oliveira et al. [18] evaluated the dispersion of carbon nanotubes (CNTs) and graphene nanoplatelets (GNPs) in an epoxy matrix using direct and indirect ultrasonication. For both systems, better electrical properties were obtained under milder dispersion conditions, whereas higher energy levels resulted in nanomaterial breakage.

Studies involving PDMS/rGO nanocomposites have demonstrated the importance of nanomaterial distribution and conductive network formation for electrical performance. Kim et al. [19] developed stretchable PDMS/rGO composites containing aligned and porous rGO structures, achieving electrical percolation at concentrations above 0.3 wt.% and demonstrating the influence of nanomaterial arrangement on electrical behavior under deformation. Similarly, Wang et al. [20] obtained PDMS/rGO nanocomposites with a three-dimensional conductive network using a latex assembly technique, enabling network formation at an rGO concentration as low as 0.44 vol.%. Surface modification has also been investigated in these systems. Jalali et al. [21] evaluated the effects of silane-functionalized rGO on the mechanical and dielectric properties of PDMS, demonstrating that the molecular structure of the silane influenced nanomaterial dispersion and charge transport. These studies indicate that the electrical properties of PDMS/rGO nanocomposites depend not only on nanomaterial concentration but also on their distribution, surface chemistry, and the formation of interconnected conductive pathways.

One approach to improve the polymer–nanomaterial interfacial interaction and reduce the need for severe mechanical dispersion conditions is the chemical functionalization of the nanomaterial surface [22,23]. Ananthasubramanian et al. [22] evaluated the effects of single-walled carbon nanotubes (SWCNTs) modified with carboxylic groups (COOH-SWCNT) and by silanization with 3-aminopropyltriethoxysilane (sily-SWCNT) in PDMS-based nanocomposites containing low reinforcement concentrations, ranging from 0.05 to 1 wt.%. The authors reported that silanization favored the interaction of the nanotubes with the silicone matrix, resulting in improved surface mechanical properties compared with nanotubes modified with carboxylic groups. Similarly, Chua et al. [24] investigated PDMS nanocomposites containing silane-functionalized MWCNTs, demonstrating that surface treatment influenced their electrical and thermal properties. Diekmann et al. [12] also investigated CNT pretreatment methods in PDMS, reporting an electrical percolation threshold of 0.1 wt.% and demonstrating that certain chemical treatments can reduce conductivity due to structural damage to the nanotubes. Silanized carbon nanomaterials have also been evaluated in other polymer matrices. Hamed Mashhadzadeh et al. [23] performed a theoretical and experimental analysis of SWCNT silanization using 3-aminopropyltriethoxysilane and its effects in epoxy, polypropylene (PP), and poly(vinyl chloride) (PVC) matrices, reporting improvements in mechanical properties associated with changes in interfacial interactions.

Although previous studies have demonstrated the potential of carbon nanomaterials and surface functionalization for improving the properties of PDMS nanocomposites, their effects depend on nanomaterial morphology, surface chemistry, and concentration. In this context, a comparative investigation of different carbon nanomaterials subjected to KH550 functionalization can contribute to understanding how surface modification influences their interaction with the polymer matrix and the development of functional properties. Thus, in this work, rGO and SWCNTs, both unmodified and functionalized with KH550, were incorporated at different concentrations into a PDMS matrix to comparatively evaluate the effects of nanomaterial morphology, concentration, and functionalization on the thermal, mechanical, and electrical properties of the nanocomposites.

## 2. Materials and Methods

### 2.1. Materials

In this work, graphite powder was purchased from Merck KGaA (Darmstadt, Germany), and single-walled carbon nanotubes (SWCNTs, Tuball) were purchased from OCSiAl, Leudelange, Luxembourg. The coupling agent γ-aminopropyltriethoxysilane (KH550) was employed for the modification of nanoparticles. For the reduction of graphene oxide and oxidation of nanotubes, commercial reagents were used: 98% sulfuric acid (H_2_SO_4_) P.A. (Neon Química, Suzano, Brazil), 65% nitric acid (HNO_3_) P.A. (Química Moderna, Santana de Parnaíba, Brazil), potassium permanganate (KMnO_4_) P.A. (Neon Química, Suzano, Brazil), 37% hydrochloric acid (HCl) P.A. (Synth Lab, Diadema, Brazil), 30% hydrogen peroxide (H_2_O_2_) P.A. (Synth Lab, Diadema, Brazil), absolute ethyl alcohol (EtOH) P.A. (Quemis, Joinville, Brazil), and acetone (CH_3_COCH_3_) P.A. (Neon Química, Suzano, Brazil). The nanocomposites were obtained using SylgardTM 184 polydimethylsiloxane elastomer base, commercialized along with the curing catalyst (Dow Chemical Company, São Paulo, Brazil). All materials and chemical reagents were used as received, without additional purification.

### 2.2. Preparation and Functionalization of rGO

Graphite powder (2 g) was added to 120 mL of H_2_SO_4_ in a 500 mL round-bottom flask and stirred for 15 min. Subsequently, 7 g of KMnO_4_ was added in small fractions to control the temperature increase caused by the exothermic reaction, and the mixture was stirred for 24 h at room temperature. The oxidation reaction was completed by slowly adding 400 mL of DI water, followed by 20 mL of 30% H_2_O_2_, while keeping the flask in an ice bath. After settling for 24 h, the material was filtered and successively washed with 400 mL of 10% HCl, 400 mL of DI water, 400 mL of EtOH, and DI water until the filtrate reached a pH of approximately 6–7. The obtained graphite oxide was exfoliated by sonication in an ultrasonic bath for 10 min and subsequently reduced in an autoclave at 120 °C and 1.25 bar for 90 min.

For functionalization, 0.2 g of the obtained rGO was dispersed in a mixture of 200 mL of EtOH and 100 mL of DI water and sonicated for 30 min. In parallel, 8 mL of γ-aminopropyltriethoxysilane was diluted in 10 mL of EtOH under stirring. The silane solution was then added to the rGO dispersion, and the mixture was stirred at 80 °C for 4 h. The functionalized material was filtered, washed with 400 mL of EtOH and excess DI water to remove residual unbound silane, and finally freeze-dried to obtain the functionalized rGO powder, hereafter denoted as rGO-KH550.

### 2.3. Oxidation and Functionalization of Carbon Nanotubes

SWCNTs (4 g) were added to a mixture of 100 mL of 98% H_2_SO_4_ and 100 mL of 65% HNO_3_ (1:1 *v*/*v*) and stirred at 100 °C for 5 h. After cooling in an ice bath, DI water corresponding to twice the total volume of the acid mixture was gradually added. The oxidized SWCNTs were filtered and washed with DI water until the filtrate reached a pH of approximately 7.

For functionalization, the oxidized SWCNTs were dispersed in 100 mL of DI water and sonicated in an ultrasonic bath for 10 min. In parallel, 10 mL of γ-aminopropyltriethoxysilane was diluted in 100 mL of EtOH under stirring. The silane solution was then added to the oxidized SWCNT dispersion, and the mixture was stirred at 80 °C for 4 h. The functionalized material was filtered, washed with EtOH followed by DI water to remove residual unbound silane, and finally freeze-dried to obtain the functionalized SWCNT powder, hereafter denoted as SWCNT-KH550.

### 2.4. Characterization of Nanomaterials

Raman spectra were acquired using a WITec alpha300 Raman microscope (WITec GmbH, Ulm, Germany) equipped with a 532 nm laser operated at a power below 5.0 mW. The wet nanomaterial samples were deposited on silicon substrates and dried in an oven at 90 °C for 30 min.

Field-emission scanning electron microscopy (FE-SEM) images were acquired using a JSM-6701F microscope (Jeol Ltd., Tokyo, Japan). The nanomaterials were deposited on silicon substrates mounted on carbon tape and analyzed at magnifications ranging from 1000× to 50,000×, without metallic coating.

X-ray photoelectron spectroscopy (XPS) spectra were acquired using a K-Alpha+ system (ThermoFisher Scientific, East Grinstead, UK) equipped with a monochromated Al Kα X-ray source operating at 1486.7 eV. Charge compensation was performed using the instrument’s dual-beam source. The XPS information depth was approximately 10 nm.

### 2.5. Preparation of Nanocomposites

The nanocomposites were prepared using a 10:1 weight ratio between the PDMS elastomeric base and the curing catalyst, with different contents of non-functionalized and functionalized nanomaterials. For this purpose, the nanomaterials were added to the PDMS, and the mixture was mechanically stirred for 40 min to promote their dispersion in the matrix. The curing catalyst was then added at the established ratio, and the mixture was stirred for an additional 5 min. Subsequently, the mixture was transferred to a PTFE mold, subjected to vacuum for 30 min, and cured at 90 °C for 24 h. rGO and rGO-KH550 were incorporated at contents of 0.5, 1.0, and 2.0 wt.%, whereas SWCNT and SWCNT-KH550 were incorporated at 0.1, 0.25, and 0.5 wt.%. The samples were identified according to the nanomaterial used and its respective content. A PDMS sample without nanomaterial addition was prepared as a reference.

### 2.6. Nanocomposite Characterization

AC conductivity, *σ_AC_*(*ω*), was evaluated using a 1260A gain-phase analyzer coupled with a 1296A dielectric interface (Solartron Analytical, Farnborough, UK). Permittivity spectra were acquired at an AC voltage of 3 V across a frequency range of 10^6^–10^−1^ Hz. Disk-shaped samples with 20 mm diameter and 1 mm thickness were coated with a thin gold layer on both faces to reduce contact resistance. Measurements were performed using a parallel-plate electrode configuration. The *σ_AC_* was calculated according to Equation (1) [6,18,25]:

(1) *σ_AC_* = *ωε*_0_*ε*″,

where *ω* is the angular frequency, *ε*_0_ is the vacuum permittivity, and *ε*″ is the imaginary part of the permittivity.

Thermogravimetric analysis (TGA) was conducted using a TA Instruments SDT-Q600 simultaneous TGA/DSC analyzer (New Castle, DE, USA). Samples were heated from 25 to 800 °C at a rate of 20 °C/min under an N_2_ atmosphere, with a gas flow rate of 60 mL/min.

Tensile tests were performed using a Z0150 universal testing machine (ZwickRoell GmbH & Co. KG, Ulm, Germany), equipped with a 10 kN load cell and operated at a crosshead speed of 50 mm/min. The specimens were prepared according to ASTM D412 [26] using Type B specimen geometry, with three specimens tested per nanocomposite formulation. Differences between successive filler concentrations were evaluated using two-tailed Welch’s *t*-test (*p* < 0.05).

Fracture surfaces of tensile-tested specimens were coated with a 10 nm gold layer and analyzed by scanning electron microscopy (SEM) using a JEOL JSM-6010LA microscope (Akishima, Japan), operating at an accelerating voltage of 10 kV.

## 3. Results and Discussion

### 3.1. Nanomaterials Surface Modification

#### 3.1.1. Surface Modification of rGO

**Figure 1:**
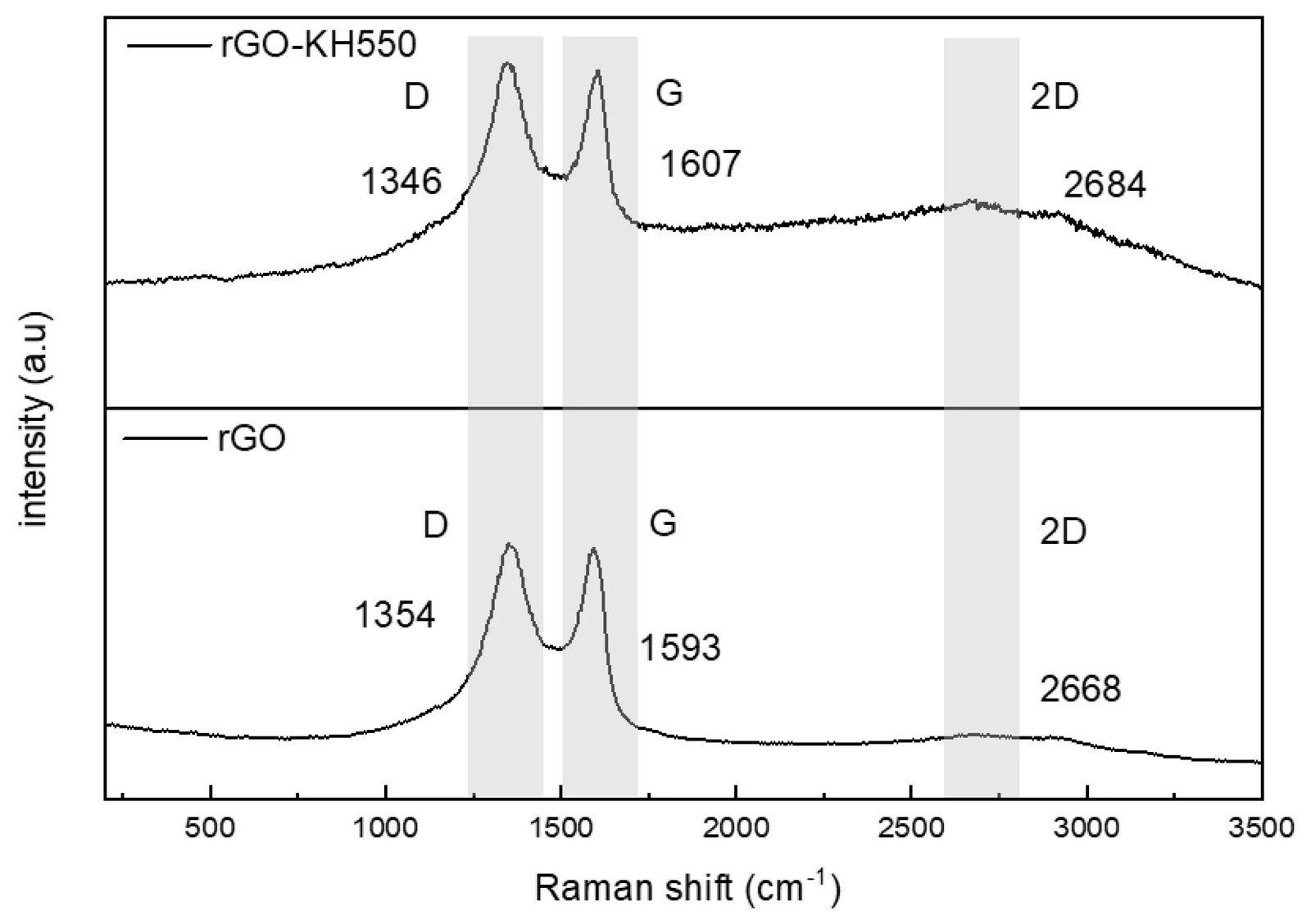
Raman spectra of rGO and rGO-KH550.

Figure 1 presents the Raman spectra of rGO and KH550-functionalized rGO samples. Both spectra display the characteristic bands of graphene: the D band (~1350 cm^−1^), associated with breathing modes of sp^2^ carbon rings activated by defects and edges; the G band (~1600 cm^−1^), related to the E_2_g stretching vibration of sp^2^ carbon atoms in the hexagonal lattice; and the 2D band (~2690 cm^−1^), corresponding to the second-order double-resonance mode, sensitive to the stacking and number of layers of the graphitic material.

The obtained Raman shift values were as follows: for rGO, the D and G bands at 1354 and 1603 cm^−1^, respectively, and for rGO-KH550, the D and G bands at 1346 and 1607 cm^−1^. A shift to a lower wavenumber of the D band and a shift to a higher wavenumber of the G band are observed after functionalization with the silane coupling agent. This modification in the vibrational profile suggests interactions between the silane groups and the sp^2^ carbon network, altering the local electronic environment. In addition, the I_D_/I_G_ ratio, calculated from the deconvolution of the bands (Table 1), was 1.06 for rGO and 1.09 for rGO-KH550. The slight increase in the I_D_/I_G_ ratio in the functionalized sample indicates the introduction of additional defects into the sp^2^ carbon lattice, consistent with the covalent anchoring or strong interactions of the silane agent on the rGO surface, which is similar to the effect induced by oxygenated groups in functionalized graphitic materials [27].

**Figure 2:**
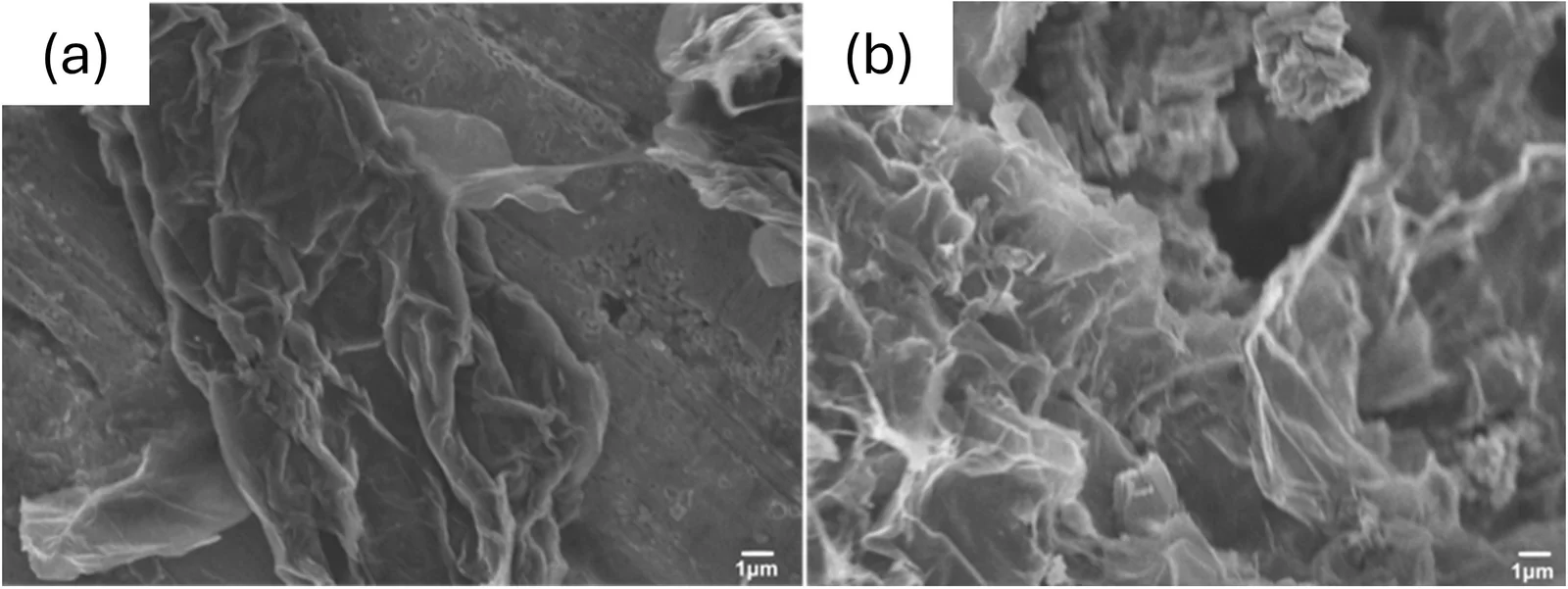
SEM images of (**a**) rGO and (**b**) rGO-KH550.

Figure 2 presents SEM images of pure rGO (a) and rGO-KH550 (b). The rGO sample exhibits an ensemble of thin, translucent sheets with lateral dimensions of several micrometers. These nanosheets appear entangled and wrinkled, a morphology characteristic of reduced graphene oxide resulting from van der Waals interactions between the lamellae and the restoration of the sp^2^ carbon network upon thermal reduction. In contrast, the rGO-KH550 sample displays a visibly rougher and more aggregated morphology. The silanized nanosheets appear thicker and more folded, with a crumpled texture covering the surface. This increased roughness and structural compaction are consistent with the grafting of KH550 onto the rGO surface, where the silane coating adds an organic layer that promotes aggregation of the sheets [27].

**Figure 3:**
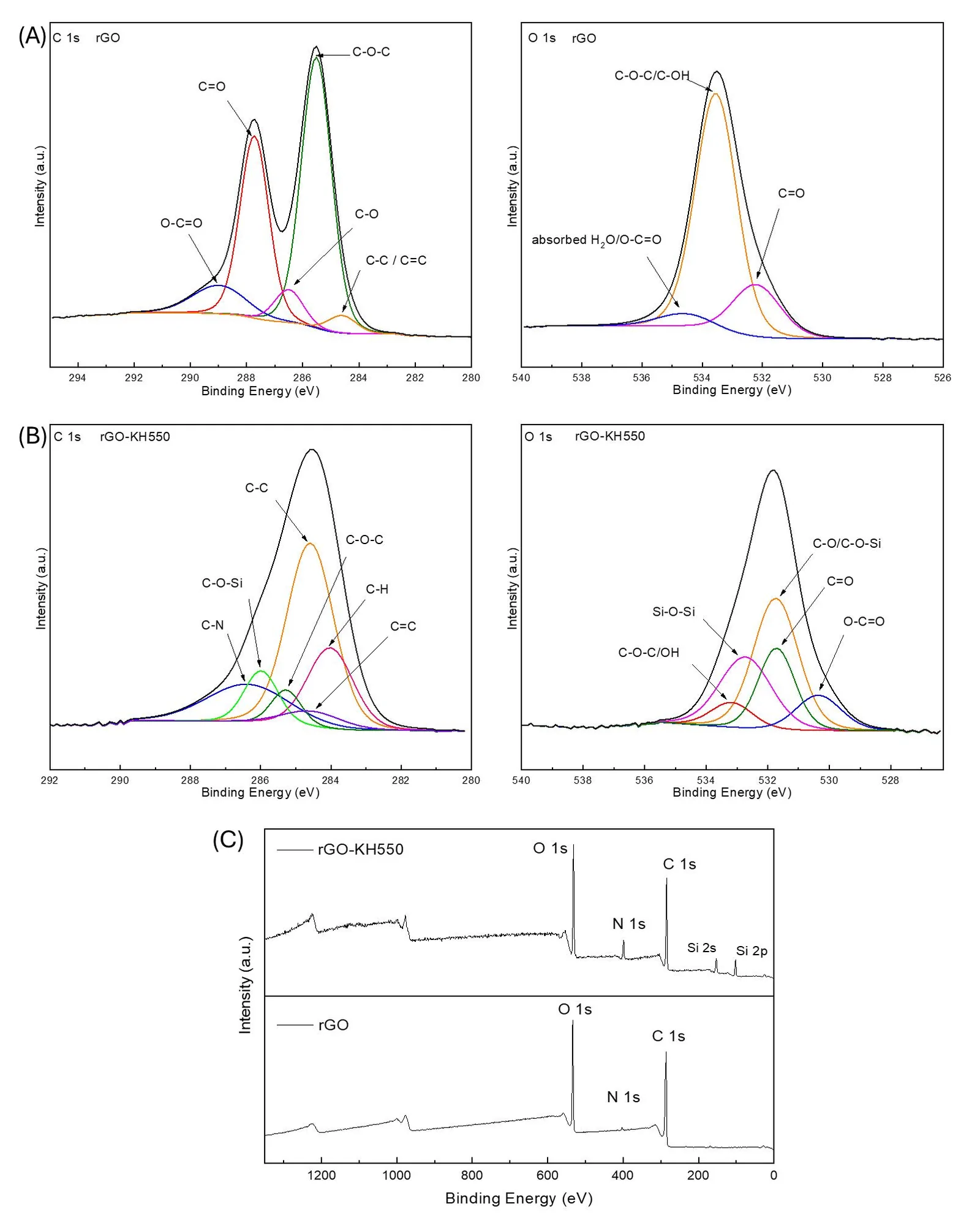
XPS of (**A**) C 1s and O 1s of rGO, (**B**) C 1s and O 1s of rGO-KH550, and (**C**) survey of rGO and rGO-KH550.

Figure 3 presents the XPS spectra of rGO and rGO-KH550. The high-resolution C 1s and O 1s deconvolution spectra for rGO (A) are shown. The carbon bond energies were assigned as follows: C–C at 284.5, C–O at 285.5, C–O–C at 286.5, C=O at 287.5, and O=C–OH at 289.0 eV [28,29]. For the oxygen bonds, the energies 531.6, 532.5, and 533.5 eV were assigned to C=O, C–O–C/C–OH, and O–C=O, respectively. Figure 3B presents the corresponding high-resolution C 1s and O 1s spectra for rGO-KH550. Following silanization, the C 1s spectrum exhibits new components at 285.9 eV (C–O–Si) and 286.3 eV (C–N), while the O 1s spectrum shows additional contributions at 532.0 eV (C–O/C–O–Si) and 532.7 eV (Si–O–Si). These spectral changes confirm effective silanization of reduced graphene oxide [30].

The increased intensity of the silicon and nitrogen signals in the rGO-KH550 survey spectrum (Figure 3C) is corroborated by the appearance of C–O–Si and Si–O–Si components in the deconvoluted C 1s and O 1s spectra. The changes in the relative contributions of C=O and C–O–C bonds between pristine and modified rGO suggest that these oxygenated functional groups participate in the silanization reaction. Furthermore, the enhanced intensity of the peak at 532.0 eV in the O 1s spectrum of rGO-KH550, associated with C–O–Si bonding, is consistent with a primary silanization route in which surface oxygenated groups react with the ethoxysilane moiety [30]. The presence of the C–N peak at 286.3 eV in the C 1s spectrum of the modified material indicates the presence of amine groups in the KH550 backbone; additionally, this feature might reflect a secondary reaction pathway in which epoxide groups on the rGO surface are ring-opened by the amine functionalities of KH550 [30]. Table 2 shows the atomic percentages of the survey spectrum of rGO and rGO-KH550.

#### 3.1.2. Surface Modification of SWCNT

**Figure 4:**
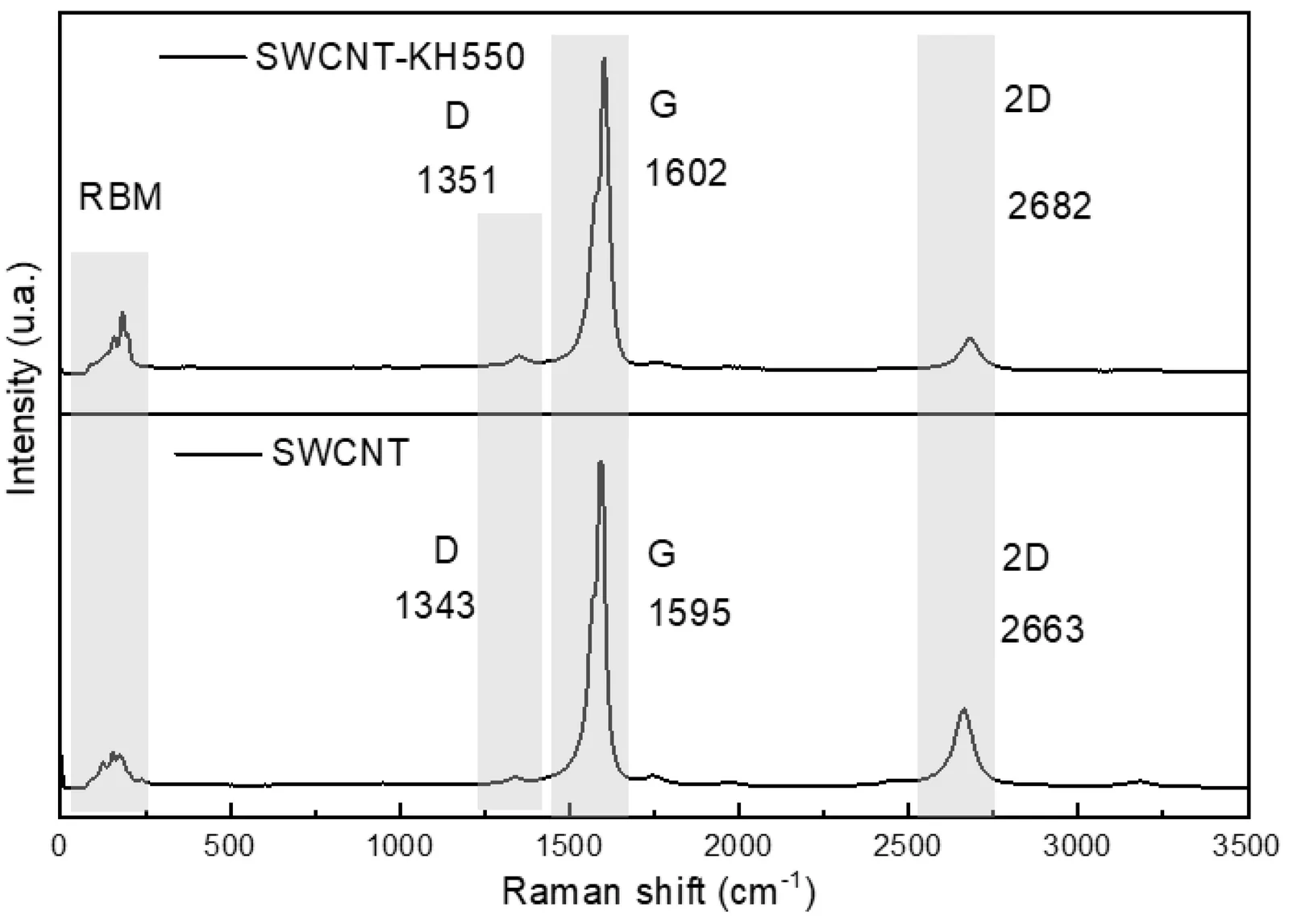
Raman spectra of SWCNT and SWCNT-KH550.

Figure 4 presents the Raman spectra of pristine SWCNT and SWCNT-KH550. Both spectra display the characteristic vibrational modes of single-walled carbon nanotubes: the radial breathing mode (RBM) at ~248 cm^−1^; the G band associated with the in-plane vibration of sp^2^-hybridized carbons (1595 cm^−1^ for SWCNT; 1602 cm^−1^ for SWCNT-KH550); the D band related to structural defects and disorder (1343 cm^−1^ for SWCNT; 1351 cm^−1^ for SWCNT-KH550); and the 2D band, the first overtone of the D band (2663 cm^−1^ for SWCNT; 2682 cm^−1^ for SWCNT-KH550).

Following silanization with KH550, upshifts (Δ) are observed in the D (~8 cm^−1^), G (~7 cm^−1^), and 2D (~19 cm^−1^) bands (Table 3). The pronounced shift in the 2D band indicates perturbation of the nanotube electronic structure due to covalent functionalization. Furthermore, the I_D_/I_G_ ratio remained essentially unchanged after functionalization (0.842 → 0.843), indicating that the modification route did not significantly disrupt the sp^2^ framework of the nanotubes—consistent with the unchanged RBM mode and with the moderate effect on the electrical conductivity of the composites at 0.25 and 0.5 wt.%, as discussed in Section 3.2.1. The band upshifts (D +8, G +7, and 2D +19 cm^−1^) and the XPS signatures (Si 2p, N 1s, C–O–Si, Si–O–Si) provide evidence for covalent functionalization [31,32]. The RBM mode remains essentially unchanged, indicating that the silanization process does not significantly alter the nanotube diameter or induce tube collapse.

**Figure 5:**
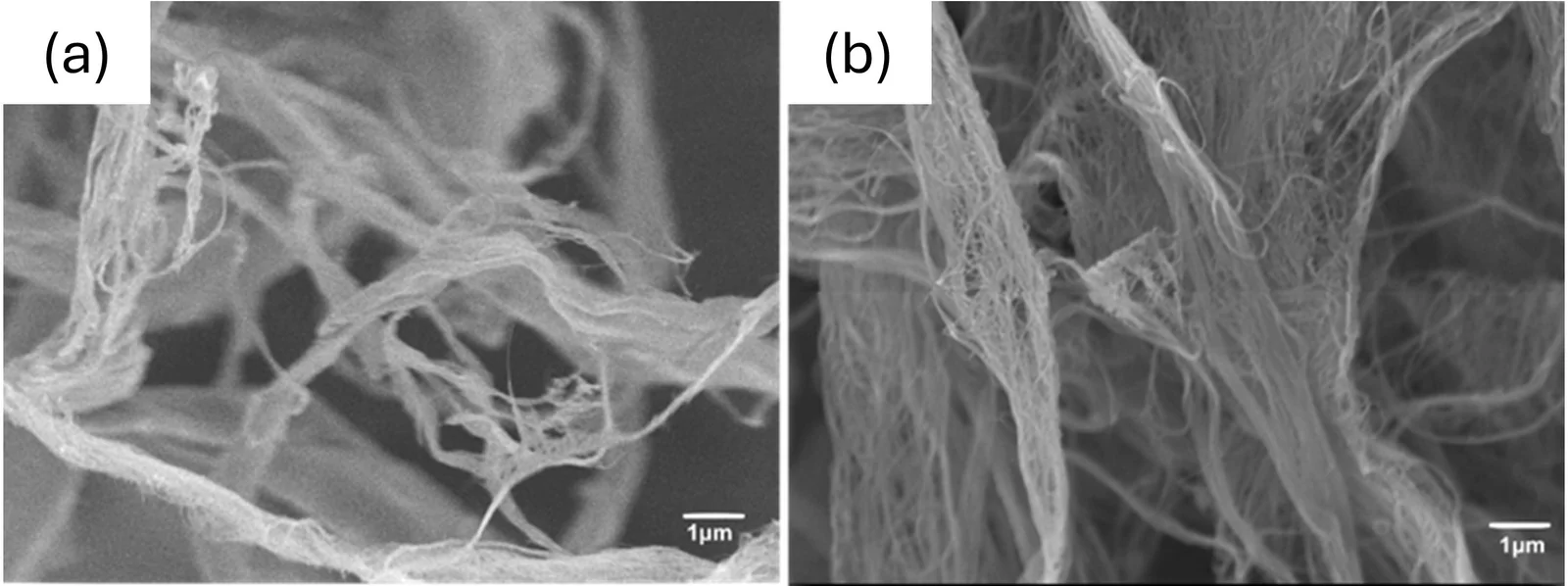
SEM of (**a**) SWCNT and (**b**) SWCNT-KH550.

Figure 5 shows scanning electron microscopy images of single-walled carbon nanotubes (SWCNTs) before and after surface modification. In their pristine state (Figure 5a), the SWCNTs appear as an entangled, web-like network of thin nanotubes with lateral dimensions of several micrometers. The individual nanotubes form an open, porous mesh with a well-defined fibrous morphology.

After silanization with KH550 (Figure 5b), the SWCNT-KH550 sample retains the overall network architecture; however, the nanotube bundles appear visibly thicker and exhibit a rougher surface texture, consistent with the grafting of the silane layer onto the nanotube walls. Notably, the long aspect ratio and structural extensions of the nanotubes are preserved without significant fragmentation. This maintained integrity is crucial, as a fragmented morphology would compromise percolation efficiency, a key requirement for effective load and charge transport through elastomeric matrices.

These observations indicate that the silanization process modifies the surface properties of the SWCNTs while preserving their structural backbone, potentially enhancing their interfacial interaction with elastomer matrices in composite applications. The preserved length and morphological integrity of the modified nanotubes underscore their potential utility in improving the mechanical and electrical properties of nanocomposites [33].

**Figure 6:**
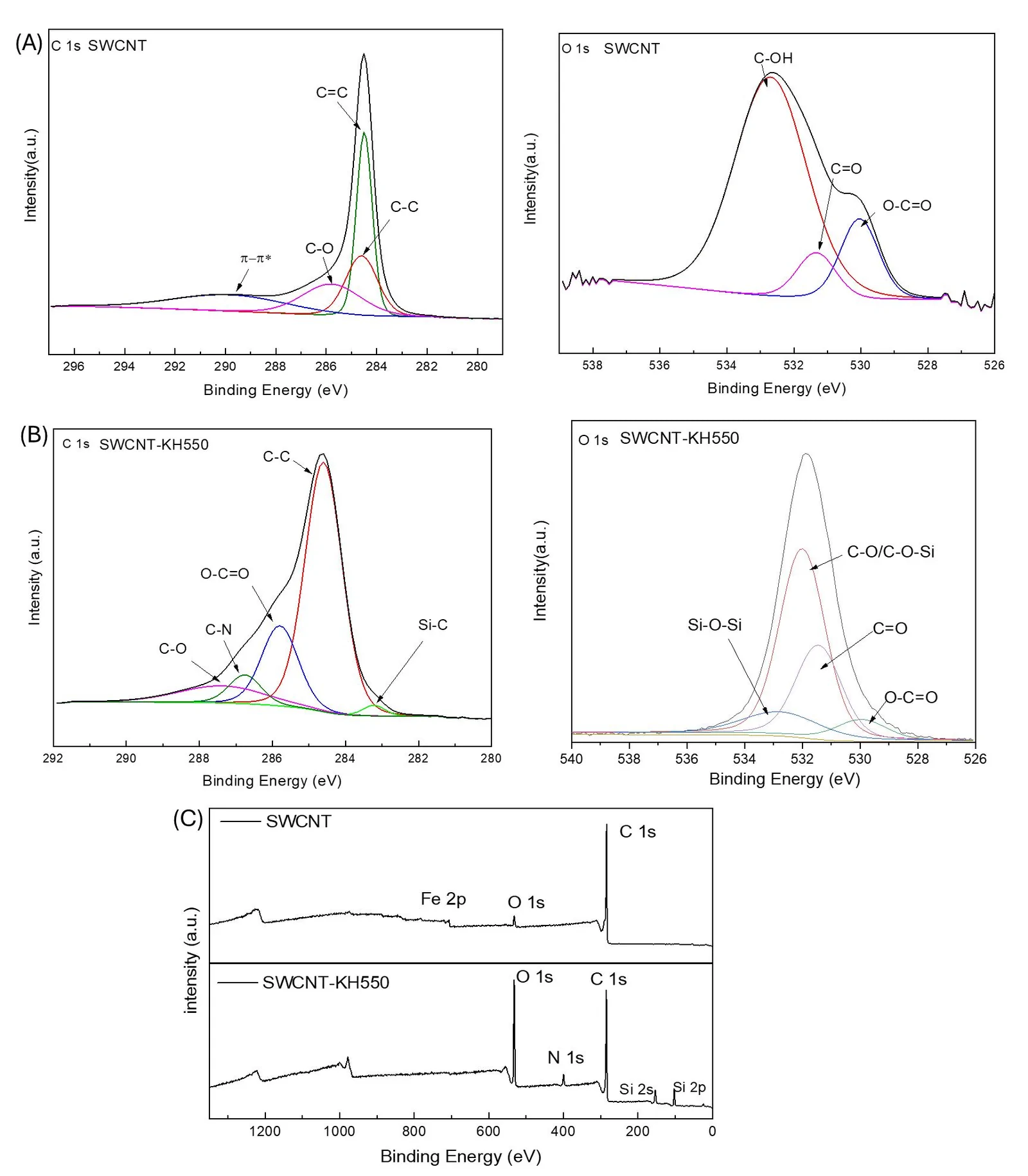
XPS of (**A**) C 1s and O 1s of SWCNT, (**B**) C 1s and O 1s of SWCNT-KH550, and (**C**) survey of SWCNT and SWCNT-KH550.

XPS analysis of the SWCNT and SWCNT-KH550 samples is presented in Figure 6. The high-resolution C 1s spectrum of pristine SWCNT (Figure 6A) was deconvoluted into contributions at 284.5 eV (C=C), 284.6 eV (C–C), and 285.78 eV (C–O). Additionally, a shake-up satellite peak (π–π*) is observed at 289.82 eV, characteristic of the sp^2^ carbon network and resulting from final-state effects upon photoelectron emission [34]. The corresponding O 1s spectrum (Figure 6A) shows components attributable to C=O, C–O, and O–C=O environments.

For SWCNT-KH550, the high-resolution C 1s spectrum (Figure 6B) confirms successful silane grafting. The deconvoluted peaks are assigned as follows: C=C at 284.5 eV, C–C at 284.6 eV, C–O at 285.8 eV, C–N at 286.7 eV, O–C=O at 287.39 eV, and Si–C at 283.2 eV [33]. The high-resolution O 1s spectrum of SWCNT-KH550 (Figure 6B) reveals additional components associated with the silane modification, including C–O/C–O–Si, Si–O–Si, and C–O–C/OH, further corroborating effective functionalization of the nanotube surface.

The survey spectrum of pristine SWCNT (Figure 6C) reveals a peak at 707.02 eV corresponding to Fe 2p, consistent with residual iron catalyst commonly employed in the CVD synthesis of carbon nanotubes [35,36]. The O 1s signal at 532.1 eV exhibits very low intensity relative to the C 1s peak at 284.6 eV, indicating minimal surface oxidation in the as-received material. Following functionalization with KH550, the survey spectrum of SWCNT-KH550 shows a marked increase in the O 1s intensity, together with the appearance of Si 2s, Si 2p, and N 1s peaks, confirming the presence of the silane coupling agent on the nanotube surface. Table 4 presents the atomic percentages of the survey spectrum of SWCNT and SWCNT-KH550. It should be noted that the silanization route comprises a preceding acid-oxidation step; therefore, the differences relative to pristine SWCNT cannot be attributed exclusively to the grafting of KH550, and the reported effects should be interpreted as the result of the complete functionalization route (oxidation followed by silanization). In addition, published results for PDMS composites containing oxidized (COOH-) and silanized SWCNTs [22] indicate that the improvements associated with silanization are not explained by the oxidation step alone.

### 3.2. Characterization of the Nanocomposites

#### 3.2.1. Electrical Properties

**Figure 7:**
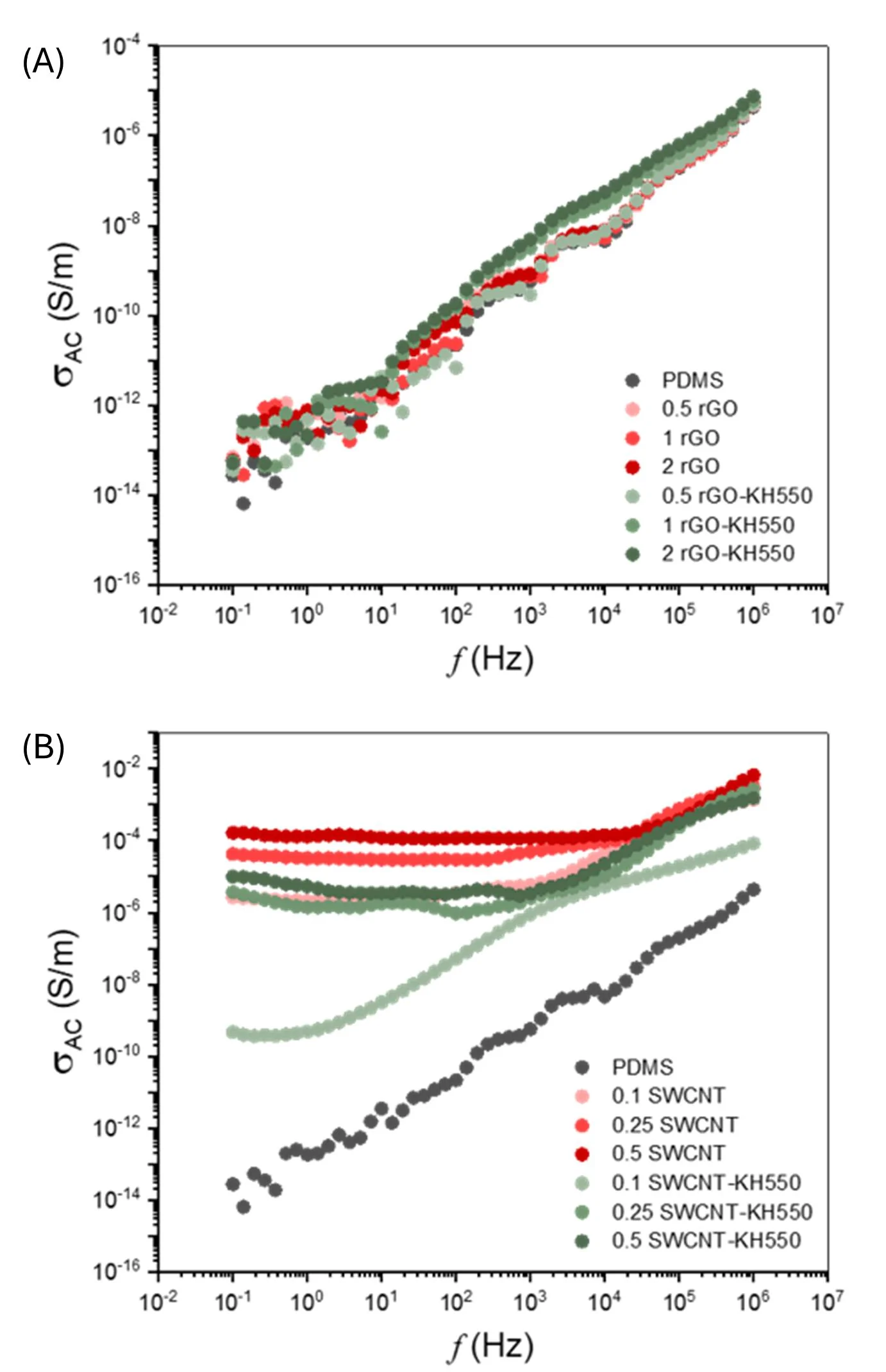
AC conductivity (σ_AC_) of nanocomposites containing (**A**) rGO and rGO-KH550 and (**B**) SWCNT and SWCNT-KH550 as a function of frequency.

Figure 7 shows the AC conductivity (σ_AC_) curves as a function of frequency. PDMS exhibits a linear frequency dependence of σ_AC_ over the entire frequency range analyzed, which is typical behavior of a dielectric material [5]. Interestingly, the incorporation of either rGO or rGO-KH550 (Figure 7A), even at the highest concentration of 2 wt.%, did not cause any significant change in the σ_AC_ behavior of the nanocomposites compared with the polymer matrix. Regarding rGO, although the reduction process partially restores the sp^2^-hybridized carbon structure, oxygen-containing groups remain on its surface, as evidenced by XPS analysis, which may impair electronic conjugation and, consequently, its intrinsic conductivity [14,15]. Additionally, the high surface area and strong interactions between rGO sheets favor their restacking and hinder dispersion within the polymer matrix, which may limit the formation of an electrically percolating network [14,37].

On the other hand, the incorporation of SWCNTs (Figure 7B), even at the lowest concentration of 0.1 wt.%, resulted in a sharp increase in conductivity. While PDMS exhibited σ_AC_ = 2.8 × 10^−14^ S/m, the 0.1 SWCNT sample reached 2.7 × 10^−6^ S/m at 0.1 Hz and exhibited a frequency-independent plateau at low frequencies, characteristic of a DC-like contribution to AC conductivity associated with charge transport through interconnected conductive pathways [25]. Increasing the SWCNT concentration further increased the conductivity, reaching 4.2 × 10^−5^ S/m and 1.7 × 10^−4^ S/m for the samples containing 0.25 and 0.5 wt.% SWCNTs, respectively. The formation of a conductive network at a concentration as low as 0.1 wt.% can be associated with the high aspect ratio of SWCNTs, which favors the establishment of contacts between nanotubes and the formation of conductive pathways throughout the polymer matrix [25], in addition to their higher intrinsic conductivity compared with rGO [38]. Capezza et al. [15] also observed significantly higher electrical conductivity for CNT-containing nanocomposites compared with those containing rGO in a natural rubber matrix, a behavior similar to that reported by Bowen et al. [38] for epoxy-based nanocomposites.

Surface modification of the SWCNTs altered their electrical conductivity behavior. The 0.1 SWCNT-KH550 sample exhibited frequency-dependent behavior, with the formation of a plateau only in the lowest-frequency decade and a σ_AC_ of 4.7 × 10^−10^ S/m at 0.1 Hz. This reduction in σ_AC_ compared with pristine SWCNTs may be associated with changes in the electrical contacts between nanotubes following functionalization, including the possible influence of the silane layer on charge transport. Punetha et al. [14] reported conductivity values of 1.7 × 10^−1^ S/m and 1.9 × 10^−8^ S/m for epoxy composites containing pristine and functionalized MWCNTs, respectively, at the same filler concentration. Diekmann et al. [12] also observed a decrease in conductivity in PDMS/CNT composites after chemical functionalization, with a value of only 7.9 × 10^−11^ S/m at 1 Hz even at a functionalized CNT content of 1 wt.%. The authors further associated this behavior with the formation of sp^3^ defects and the reduction in nanotube length resulting from the modification process.

On the other hand, it is interesting to note that increasing the SWCNT-KH550 concentration led to an increase in σ_AC_, following a trend similar to that observed for pristine SWCNTs. The samples containing 0.25 and 0.5 wt.% SWCNT-KH550 exhibited σ_AC_ values of 3.6 × 10^−6^ S/m and 1.0 × 10^−5^ S/m, respectively, only about one order of magnitude lower than those of the corresponding pristine SWCNT samples. This behavior indicates that increasing the SWCNT-KH550 concentration favors the establishment of contacts between nanotubes and the formation of a conductive network, partially compensating for the negative effects resulting from surface modification. In addition, the small variation in the I_D_/I_G_ ratio observed by Raman spectroscopy (Table 3) suggests that the carbon structure of the SWCNTs was partially preserved after modification.

#### 3.2.2. Thermal Properties

**Figure 8:**
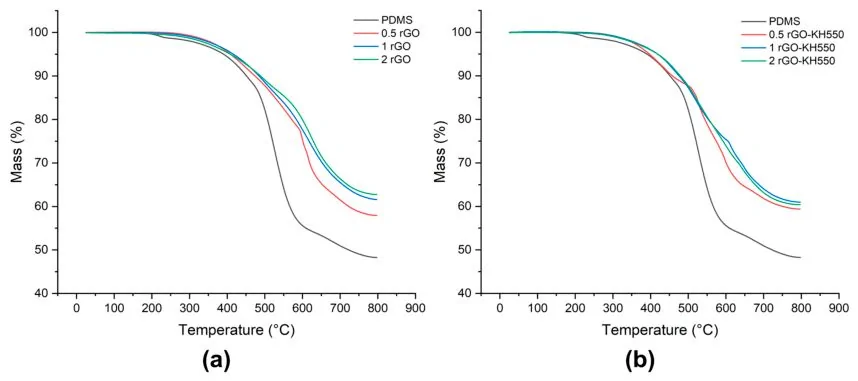
Thermogravimetric analysis of neat PDMS and PDMS composites filled with (**a**) rGO and (**b**) rGO-KH550.

Figure 8 presents the thermogravimetric curves of neat PDMS and the nanocomposites containing rGO and rGO-KH550. The main degradation temperature was determined from the corresponding DTG peak maximum.

All samples exhibited three distinct mass-loss events. For neat PDMS, these occurred at approximately 220 °C, 453 °C, and 526 °C. The first mass loss, at a relatively low temperature, is associated with the removal of low-molecular-weight volatile species. The second stage, centered at approximately 453 °C, relates to initial cleavage and rearrangement of the PDMS network, while the main degradation step at 526 °C is associated with extensive decomposition of the polymer chains and the release of volatile siloxane products [39].

The incorporation of rGO markedly modified the thermal degradation profile of PDMS. Main degradation temperatures increased to 615 °C, 614 °C, and 621 °C for 0.5, 1, and 2 wt.% rGO, respectively, an increase of approximately 90 °C relative to neat PDMS. This stabilization is associated with the high thermal stability of rGO and its barrier effect, which hinders diffusion and release of volatile products generated during degradation [40,41]. Interactions between rGO and the polymer matrix may also restrict chain mobility at the interfacial region, making cleavage and volatilization of degradation products more difficult. The higher residual masses observed for the composites, compared with neat PDMS, reflect the contribution of the thermally stable carbonaceous phase [40].

Silane functionalization further modified the thermal degradation profiles of the rGO-containing composites, introducing an additional mass-loss event between 439 °C and 518 °C. This event can be attributed to the decomposition of organic groups introduced during silanization [42], consistent with the presence of Si- and N-containing species identified by XPS. The lower residual masses of the rGO-KH550 composites compared with those containing unmodified rGO are also consistent with changes in composition and thermal degradation behavior following functionalization.

**Figure 9:**
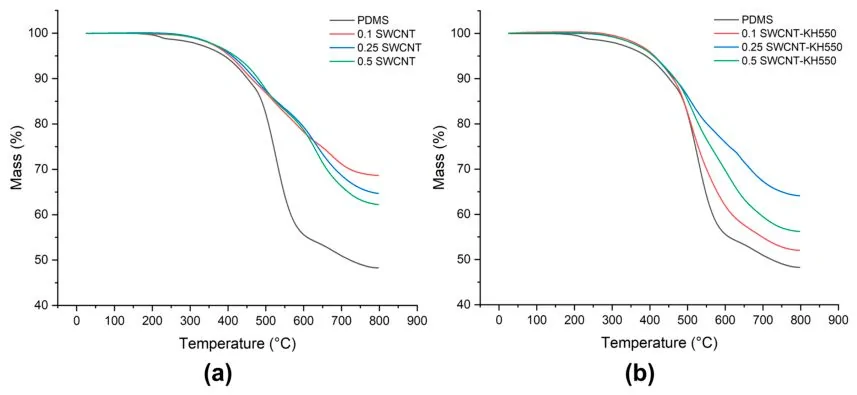
Thermogravimetric analysis of neat PDMS and PDMS composites filled with (**a**) SWCNT and (**b**) SWCNT-KH550.

Figure 9 presents the thermogravimetric curves of neat PDMS and the nanocomposites containing SWCNT and SWCNT-KH550.

The incorporation of SWCNTs also increased the main degradation temperature of PDMS, although the magnitude of this increase varied with filler content. The highest value, 672 °C, was observed for the composite containing 0.1 wt.% SWCNT, while the values for 0.25 and 0.5 wt.% SWCNT were 632 and 633 °C, respectively. The high aspect ratio of SWCNT may contribute to enhanced thermal stability by hindering the transport of volatile degradation products [43,44]. However, the stabilization effect was not proportional to SWCNT concentration, with higher loadings resulting in lower and nearly identical degradation temperatures. This non-monotonic behavior may reflect the combined influence of filler distribution and interfacial interactions, while residual metallic species may also promote catalytic degradation reactions, as previously reported for CNT/silicone composites [45].

KH550 functionalization also altered the thermal degradation behavior of the SWCNT-containing composites. An additional mass-loss event appeared between 427 and 443 °C, likely associated with the decomposition of the organic groups introduced during silanization [42]. The main degradation temperatures were 510, 641, and 611 °C for composites containing 0.1, 0.25, and 0.5 wt.% SWCNT-KH550, respectively. Compared with their unmodified counterparts, functionalized composites exhibited lower residual masses. Functionalization also affected the main degradation temperature differently depending on filler content, resulting in a substantial decrease at 0.1 wt.%, a slight increase at 0.25 wt.%, and a decrease at 0.5 wt.%.

#### 3.2.3. Mechanical Properties

The tensile properties of neat PDMS and the corresponding nanocomposites are presented in Table 5 and Table 6. Neat PDMS exhibited an ultimate tensile strength of 2.9 ± 0.7 MPa and an elongation at break of 72 ± 11%, which were used as reference values for evaluating the effects of rGO, SWCNT, and KH550 functionalization.

The tensile properties of the rGO-containing nanocomposites varied with filler content. The ultimate tensile strength increased from 2.4 ± 0.2 MPa at 0.5 wt.% rGO to 3.1 ± 0.1 MPa at 1 wt.%, then slightly declined to 2.9 ± 0.9 MPa at 2 wt.%. Meanwhile, elongation at break increased from 68 ± 9% to 96 ± 9% and 100 ± 17%, respectively. These results indicate that rGO incorporation primarily favored elongation at break, particularly at 1 and 2 wt.%, while tensile strength did not exhibit a consistent trend with filler content. However, the relatively large variability observed at 2 wt.% rGO, particularly in tensile strength, limits the interpretation of differences among formulations.

In contrast, the SWCNT-containing composites exhibited progressively lower tensile strength and elongation at break with increasing filler content. Between 0.1 and 0.5 wt.%, the ultimate tensile strength decreased from 2.4 ± 0.6 to 1.4 ± 0.1 MPa, while elongation at break dropped from 59 ± 15% to 44 ± 4%. Although the high aspect ratio of SWCNTs can favor stress transfer, effective reinforcement also depends on filler–matrix interfacial adhesion and dispersion. Huang et al. [46] reported that CNT-rich regions in PDMS can act as stress concentration sites, promoting crack initiation and propagation, which could account for the deterioration of tensile properties at higher SWCNT loadings.

Overall, the rGO and SWCNT composites exhibited distinct responses to increasing filler content. While rGO incorporation generally favored higher elongation at break at concentrations of 1 and 2 wt.%, increasing SWCNT content was associated with a progressive reduction in both tensile strength and elongation at break.

Silane functionalization of carbon nanotubes has been reported to improve the mechanical response of PDMS composites. Chua et al. [24] attributed the improved properties of silanized MWCNTs, relative to unmodified nanotubes, to enhanced dispersion in the matrix, while Ananthasubramanian et al. [22] related the superior performance of silanized SWCNTs, relative to carboxylated SWCNTs, to Si–O–Si bonding with the silicone matrix and reduced agglomeration. Similarly, KH550 functionalization may improve the compatibility of carbon nanomaterials with PDMS, favoring dispersion, interfacial adhesion, and stress transfer [46,47].

For the rGO-containing composites, KH550 functionalization was associated with a decreasing trend in tensile strength with increasing filler content, while elongation at break remained higher than that of neat PDMS. At 0.5 wt.%, the functionalized composite exhibited higher mean values of both properties than its unmodified counterpart, although the tensile strength showed considerable variability. At higher concentrations, the effects of functionalization were less consistent. On the other hand, SWCNT-KH550 at 0.25 and 0.5 wt.% exhibited higher ultimate tensile strengths (2.7 and 3.2 MPa, respectively) and elongations at break (71 and 67%, respectively) than the corresponding pristine SWCNT composites (2.1 and 1.4 MPa, and 48 and 44%, respectively). These improvements suggest that functionalization enhanced filler–matrix interactions, contributing to the improved tensile performance of the nanocomposites. However, the effect of KH550 functionalization depended on the SWCNT loading, as both tensile strength and elongation at break were lower for SWCNT-KH550 than for pristine SWCNT at 0.1 wt.%.

The results indicate that KH550 functionalization influenced the tensile behavior of the nanocomposites differently depending on filler type and concentration. Although higher mean values of both properties were observed at 0.5 wt.% rGO-KH550 and at 0.25 and 0.5 wt.% SWCNT-KH550 relative to their unmodified counterparts, these effects were not consistent across all formulations. Notably, SWCNT-KH550 at 0.25 and 0.5 wt.% exhibited tensile properties approaching those of neat PDMS, suggesting that functionalization partially mitigated the deterioration observed with unmodified SWCNTs.

#### 3.2.4. Morphological Characterization

**Figure 10:**
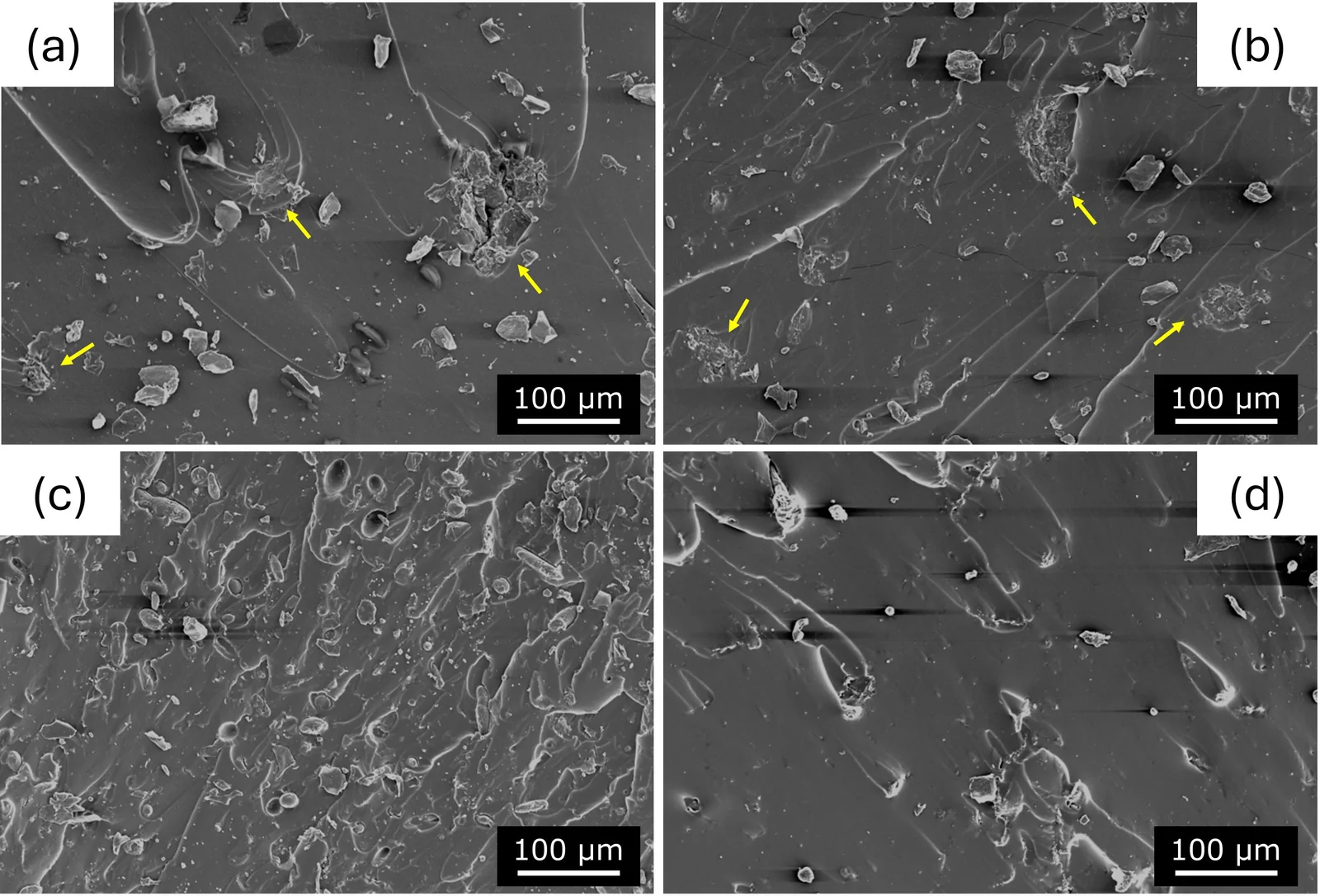
SEM micrographs of the tensile fracture surfaces of PDMS nanocomposites containing (**a**) 2 rGO, (**b**) 2 rGO-KH550, (**c**) 0.5 SWCNT, and (**d**) 0.5 SWCNT-KH550. Yellow arrows indicate regions of apparent agglomeration.

Figure 10 presents SEM micrographs of the tensile fracture surfaces of the nanocomposites containing the highest concentrations of each nanomaterial. The 2 rGO sample (Figure 10a) exhibits regions of apparent agglomeration (indicated by yellow arrows), with dimensions of up to approximately 80 µm, heterogeneously distributed within the PDMS matrix. The formation of these agglomerates may be associated with interactions between residual functional groups on the rGO sheets, favoring their association, particularly at higher concentrations [46]. Following KH550 functionalization (2 rGO-KH550, Figure 10b), a relatively more homogeneous fracture surface is observed, with fewer regions of apparent agglomeration. Although functionalization reduced the occurrence of these structures, their residual presence may have limited stress transfer between rGO and the PDMS matrix, contributing to the lack of consistent improvements in mechanical properties, as discussed in Section 3.2.2.

On the other hand, the nanocomposite containing 0.5 wt.% SWCNT (Figure 10c) exhibits numerous bright spots distributed across the fracture surface within the PDMS matrix, possibly associated with the presence of nanotubes or small aggregates [43]. After KH550 functionalization (Figure 10d), a more homogeneous surface is observed, with fewer of these features, suggesting a more uniform distribution of nanotubes, possibly favored by improved interactions with the PDMS matrix. This interpretation is consistent with the higher mean tensile strength and elongation at break observed for the functionalized nanocomposite compared with its unmodified counterpart. These observations suggest that nanofiller type and morphology, along with surface chemistry, influence interfacial interactions and contribute to the distinct mechanical responses following functionalization.

## 4. Conclusions

The silane functionalization route effectively modified the surface chemistry of both rGO and SWCNTs, as mainly evidenced by XPS analysis, with complementary changes observed by Raman spectroscopy and SEM. The incorporation of the two carbon nanomaterials resulted in markedly different responses in PDMS nanocomposites. rGO did not significantly alter the electrical conductivity of PDMS at concentrations up to 2.0 wt.%, whereas SWCNTs promoted the formation of conductive pathways at concentrations as low as 0.1 wt.%. Silane functionalization reduced the electrical conductivity of the SWCNT-containing nanocomposites, particularly at the lowest concentration, although increasing the SWCNT-KH550 content partially compensated for this effect. Both nanomaterials modified the thermal degradation behavior of PDMS, while the mechanical response depended on nanomaterial morphology, concentration, and surface modification. Functionalization had a more pronounced effect on the mechanical response of SWCNT-containing nanocomposites than on those containing rGO. Overall, the results demonstrate that the effects of silane functionalization cannot be considered independently of nanomaterial morphology and concentration. The development of functional PDMS nanocomposites therefore requires balancing nanomaterial–matrix interactions with the preservation and formation of conductive networks according to the targeted properties.

## Figures and Tables

**Table 1:** I_D_/I_G_ ratio from the intensity of GO and rGO-KH550.

Samples	Intensity		I_D_/I_G_
	D	G	
rGO	2110	1990	1.06
rGO-KH550	3515	3199	1.09

**Table 2:** Survey XPS atomic percentages of GO and rGO-KH550.

Sample	C 1s	O 1s	N 1s	Si 2p	S 2p
rGO	75.31	24.69	—	—	—
rGO-KH550	55.06	26.77	7.03	11.14	—

**Table 3:** I_D_/I_G_ ratio and Δ (cm^−1^) from the intensity of SWCNT and SWCNT-KH550.

Samples	Intensity			Δ (cm^−1^)			I_D_/I_G_
	D	G	2D	D	G	2D	
SWCNT	1343	1595	2663	7	8	19	0.842
SWCNT-KH550	1351	1602	2682				0.843

**Table 4:** Survey XPS atomic percentages (%) of SWCNT and SWCNT-KH550.

Sample	C 1s	O 1s	N 1s	Si 2p	S 2p	Fe 2p
SWCNT	82.08	10.43	—	—	—	7.49
SWCNT-KH550	71.94	25.50	0.64	0.60	0.30	0.01

**Table 5:** Tensile properties of neat PDMS and PDMS composites containing rGO and rGO-KH550.

Samples	Ultimate Tensile Strength (MPa)	Elongation at Break (%)
PDMS	2.9 ± 0.7	72 ± 11
0.5 rGO	2.4 ± 0.2	68 ± 9
1 rGO	3.1 ± 0.1 *	96 ± 9 *
2 rGO	2.9 ± 0.9	100 ± 17
0.5 rGO-KH550	3.2 ± 1.2	94 ± 3
1 rGO-KH550	2.8 ± 0.2	89 ± 10
2 rGO-KH550	2.5 ± 0.4	110 ± 22

Note: * indicates *p* < 0.05.

**Table 6:** Tensile properties of neat PDMS and PDMS composites containing SWCNT and SWCNT-KH550.

Samples	Ultimate Tensile Strength (MPa)	Elongation at Break (%)
PDMS	2.9 ± 0.7	72 ± 11
0.1 SWCNT	2.4 ± 0.6	59 ± 15
0.25 SWCNT	2.1 ± 0.5	48 ± 11
0.5 SWCNT	1.4 ± 0.1	44 ± 4
0.1 SWCNT-KH550	1.6 ± 0.1	44 ± 2 *
0.25 SWCNT-KH550	2.7 ± 0.2 *	71 ± 9 *
0.5 SWCNT-KH550	3.2 ± 0.1 *	67 ± 13

Note: * indicates *p* < 0.05.

## Data Availability

The data that support the findings of this study are available from the corresponding author upon reasonable request.

## References

[1] Bokobza L. (2023). Elastomer Nanocomposites: Effect of Filler–Matrix and Filler–Filler Interactions. Polymers.

[2] Kim T.G., Eom H.S., Kim J.H., Jung J.K., Jang K., Lee S.J. (2021). Electrically Conductive Silicone-Based Nanocomposites Incorporated with Carbon Nanotubes and Silver Nanowires for Stretchable Electrodes. ACS Omega.

[3] de Sousa Junior R.R., Garcia G.E.S., Antonino L.D., Gouveia J.R., dos Santos D.J., Carastan D.J. (2024). Dielectric Elastomers Based on SEBS Gel: The Impact of Adding Kraft Lignin on Electro-Mechanical Performance. Express Polym. Lett..

[4] Wiranata A., Ishii Y., Hosoya N., Maeda S. (2021). Simple and Reliable Fabrication Method for Polydimethylsiloxane Dielectric Elastomer Actuators Using Carbon Nanotube Powder Electrodes. Adv. Eng. Mater..

[5] de Sousa R.R., Sacramento J.B., da Silva L.C.E., Becker D., Vidotti S.E., Carastan D.J. (2023). High-Performance Block-Copolymer-Based Dielectric Elastomers with Enhanced Mechanical Properties. ACS Appl. Polym. Mater..

[6] de Sousa R.R., Heinze D.A., Sacramento J.B., Lanfredi A.J.C., Carastan D.J. (2023). Electrical Conductivity and In Situ SAXS Probing of Block Copolymer Nanocomposites Under Mechanical Stretching. ACS Appl. Mater. Interfaces.

[7] Kumar V., Alam M.N., Park S. (2024). Review on Functionalized CNTs Reinforced Silicone Rubber Composites for Potential Wearable Applications. Polym. Compos..

[8] Kumar V., Parvin N., Joo S.W., Mandal T.K., Park S.S. (2025). Great Carbon Nano Materials Based Composites for Electronic Skin: Intelligent Sensing, and Self-Powered Nano Generators. Nano Energy.

[9] Perumalla A., Thomas T., Kalpathy S.K. (2024). Metal Oxide-Dispersed PDMS Nanocomposite Films with Enhanced Heat-Shielding Ability and Optical Transparency. Opt. Mater..

[10] Zhang F., Sun Y., Guo L., Zhang Y., Liu D., Feng W., Shen X., Zheng Q. (2024). Microstructural Welding Engineering of Carbon Nanotube/Polydimethylsiloxane Nanocomposites with Improved Interfacial Thermal Transport. Adv. Funct. Mater..

[11] Kumar V., Alam M.N., Manikkavel A., Song M., Lee D., Park S. (2021). Silicone Rubber Composites Reinforced by Carbon Nanofillers and Their Hybrids for Various Applications: A Review. Polymers.

[12] Diekmann A., Omelan M.C.V., Giese U. (2021). Influence of Carbon Nanotube-Pretreatment on the Properties of Polydimethylsiloxane/Carbon Nanotube-Nanocomposites. Polymers.

[13] Azam S., Kumar V., Park S. (2024). Electro-bio-mechanical Behavior of Graphite Nanoplatelets–Silicone Rubber-based Composites for Wearable Electronic Systems. J. Appl. Polym. Sci..

[14] Punetha V.D., Rana S., Yoo H.J., Chaurasia A., McLeskey J.T., Ramasamy M.S., Sahoo N.G., Cho J.W. (2017). Functionalization of Carbon Nanomaterials for Advanced Polymer Nanocomposites: A Comparison Study between CNT and Graphene. Prog. Polym. Sci..

[15] Capezza A., Andersson R.L., Ström V., Wu Q., Sacchi B., Farris S., Hedenqvist M.S., Olsson R.T. (2019). Preparation and Comparison of Reduced Graphene Oxide and Carbon Nanotubes as Fillers in Conductive Natural Rubber for Flexible Electronics. ACS Omega.

[16] Nioua Y., El Bouazzaoui S., Melo B.M.G., Prezas P.R.S., Graça M.P.F., Achour M.E., Costa L.C., Brosseau C. (2017). Analyzing the Frequency and Temperature Dependences of the Ac Conductivity and Dielectric Analysis of Reduced Graphene Oxide/Epoxy Polymer Nanocomposites. J. Mater. Sci..

[17] Lavagna L., Nisticò R., Musso S., Pavese M. (2021). Functionalization as a Way to Enhance Dispersion of Carbon Nanotubes in Matrices: A Review. Mater. Today Chem..

[18] de Oliveira M.M., Forsberg S., Selegård L., Carastan D.J. (2021). The Influence of Sonication Processing Conditions on Electrical and Mechanical Properties of Single and Hybrid Epoxy Nanocomposites Filled with Carbon Nanoparticles. Polymers.

[19] Kim T.A., Pyo J.B., Lee S., Park M. (2019). Highly Aligned and Porous Reduced Graphene Oxide Structures and Their Application for Stretchable Conductors. J. Ind. Eng. Chem..

[20] Wang X., Tang Y., Cheng S., Gao Q., Yuan Y., Li A., Guan S. (2022). PDMS-Based Conductive Elastomeric Composite with 3D Reduced Graphene Oxide Conductive Network for Flexible Strain Sensor. Compos. Part A Appl. Sci. Manuf..

[21] Jalali S., Khakbaz F., Razzaghi-Kashani M. (2026). Mechanistic Evaluation of Silane Spacer Length on Mechanical and Dielectric Properties of Polydimethylsiloxane-Graphene Nanocomposites. Polym. Eng. Sci..

[22] Ananthasubramanian P., Sahay R., Raghavan N. (2024). Investigation of the Surface Mechanical Properties of Functionalized Single-Walled Carbon Nanotube (SWCNT) Reinforced PDMS Nanocomposites Using Nanoindentation Analysis. RSC Adv..

[23] Hamed Mashhadzadeh A., Fereidoon A., Ghorbanzadeh Ahangari M. (2017). Surface Modification of Carbon Nanotubes Using 3-Aminopropyltriethoxysilane to Improve Mechanical Properties of Nanocomposite Based Polymer Matrix: Experimental and Density Functional Theory Study. Appl. Surf. Sci..

[24] Chua T.P., Mariatti M., Azizan A., Rashid A.A. (2010). Effects of Surface-Functionalized Multi-Walled Carbon Nanotubes on the Properties of Poly(Dimethyl Siloxane) Nanocomposites. Compos. Sci. Technol..

[25] Mergen Ö.B., Umut E., Arda E., Kara S. (2020). A Comparative Study on the AC/DC Conductivity, Dielectric and Optical Properties of Polystyrene/Graphene Nanoplatelets (PS/GNP) and Multi-Walled Carbon Nanotube (PS/MWCNT) Nanocomposites. Polym. Test..

[26] (2021). Standard Test Methods for Vulcanized Rubber and Thermoplastic Elastomers.

[27] Ayora-Gutiérrez G., Abreu-Rejón A.D., May-Pat A., Guerrero-Bermea C., Fernández-Escamilla V.V., Rodríguez-Fuentes N., Cervantes-Uc J.M., Uribe-Calderon J.A. (2024). Effect of Surface Modification of Graphene Oxide with a Reactive Silane Coupling Agent on the Mechanical Properties and Biocompatibility of Acrylic Bone Cements. J. Biomater. Sci. Polym. Ed..

[28] Albers P.W., Leich V., Ramirez-Cuesta A.J., Cheng Y., Hönig J., Parker S.F. (2022). The Characterisation of Commercial 2D Carbons: Graphene, Graphene Oxide and Reduced Graphene Oxide. Mater. Adv..

[29] Chen X., Wang X., Fang D. (2020). A Review on C1s XPS-Spectra for Some Kinds of Carbon Materials. Fuller. Nanotub. Carbon Nanostructures.

[30] Abbas S.S., Rees G.J., Kelly N.L., Dancer C.E.J., Hanna J.V., McNally T. (2018). Facile Silane Functionalization of Graphene Oxide. Nanoscale.

[31] Dresselhaus M.S., Dresselhaus G., Jorio A., Souza Filho A.G., Saito R. (2002). Raman Spectroscopy on Isolated Single Wall Carbon Nanotubes. Carbon.

[32] Saito R., Hung N.T., Yang T., Huang J., Liu H., Gulo D.P., Han S., Tong L. (2025). Deep-Ultraviolet and Helicity-Dependent Raman Spectroscopy for Carbon Nanotubes and 2D Materials. Small.

[33] Manoharan G., Bösel P., Thien J., Holtmannspötter M., Meingast L., Schmidt M., Eickmeier H., Haase M., Maultzsch J., Steinhart M. (2023). Click-Functionalization of Silanized Carbon Nanotubes: From Inorganic Heterostructures to Biosensing Nanohybrids. Molecules.

[34] Araujo J.R., Silva A.M., Gouvêa C.P., Lopes E.S., Santos R.A.A., Terrazos L.A., Capaz R.B., Achete C.A., Maciel I.O. (2016). Phosphorous Bonding in Single Wall Carbon Nanotubes Studied by X-Ray Photoelectron Spectroscopy and DFT Calculations. Carbon.

[35] Merkel L., Setaro A., Halbig C.E., Shimizu S., Yoshii T., Nishihara H., Hilal T., Algara-Siller G., Koch C., Eigler S. (2024). Structural Model of Oxidatively Unzipped Narrow Single-Walled Carbon Nanotubes. Carbon.

[36] Li Z., Sun Y., Hu F., Liu D., Zhang X., Ren J., Guo H., Shalash M., He M., Hou H. (2025). An Overview of Polymer-Based Thermally Conductive Functional Materials. J. Mater. Sci. Technol..

[37] Roy D., B V., Mandal S., Gupta A., Sochor B., Koyiloth Vayalil S., Kraus T. (2025). Origin of Dynamic Network Formation of 2D Nanofillers in a Flexible Matrix. Small Struct..

[38] Bowen C.R., Buschhorn S., Adamaki V. (2014). Manufacture and Characterization of Conductor-Insulator Composites Based on Carbon Nanotubes and Thermally Reduced Graphene Oxide. Pure Appl. Chem..

[39] Camino G., Lomakin S., Lageard M. (2002). Thermal Polydimethylsiloxane Degradation. Part 2. The Degradation Mechanisms. Polymer.

[40] Sarath P., Moni G., George J.J., Haponiuk J.T., Thomas S., George S.C. (2021). A Study on the Influence of Reduced Graphene Oxide on the Mechanical, Dynamic Mechanical and Tribological Properties of Silicone Rubber Nanocomposites. J. Compos. Mater..

[41] Łapińska A., Grochowska N., Antonowicz J., Michalski P., Dydek K., Dużyńska A., Daniszewska A., Ojrzyńska M., Zeranska K., Zdrojek M. (2022). Influence of the Filler Distribution on PDMS-Graphene Based Nanocomposites Selected Properties. Sci. Rep..

[42] Wang X., Chen D., Zhong W., Zhang L., Fan X., Cai Z., Zhu M. (2020). Experimental and Theoretical Evaluations of the Interfacial Interaction between Carbon Nanotubes and Carboxylated Butadiene Nitrile Rubber: Mechanical and Damping Properties. Mater. Des..

[43] Shang S., Gan L., Yuen M.C., Jiang S., Luo N.M. (2014). Carbon Nanotubes Based High Temperature Vulcanized Silicone Rubber Nanocomposite with Excellent Elasticity and Electrical Properties. Compos. Part A Appl. Sci. Manuf..

[44] Flagg D.H., McCarthy T.J. (2019). Carbon Nanotubes Readily Disperse in Linear Silicones and Improve the Thermal Stability of Dimethylsilicone Elastomers. Langmuir.

[45] Li Z., Lin W., Moon K.-S., Wilkins S.J., Yao Y., Watkins K., Morato L., Wong C. (2011). Metal Catalyst Residues in Carbon Nanotubes Decrease the Thermal Stability of Carbon Nanotube/Silicone Composites. Carbon.

[46] Huang N., Zang J., Zhang G., Guan L., Li S., Zhao L., Tang L. (2017). Efficient Interfacial Interaction for Improving Mechanical Properties of Polydimethylsiloxane Nanocomposites Filled with Low Content of Graphene Oxide Nanoribbons. RSC Adv..

[47] Bai L., Li Z., Zhao S., Zheng J. (2018). Covalent Functionalization of Carbon Nanotubes with Hydroxyl-Terminated Polydimethylsiloxane to Enhance Filler Dispersion, Interfacial Adhesion and Performance of Poly(Methylphenylsiloxane) Composites. Compos. Sci. Technol..

